# Adaptation and validation of a German version of the Dickman impulsivity inventory for the assessment of functional and dysfunctional impulsivity

**DOI:** 10.1038/s41598-021-02775-1

**Published:** 2021-12-02

**Authors:** Philippa Hüpen, Alina T. Henn, Ute Habel

**Affiliations:** 1 Department of Psychiatry, Psychotherapy and Psychosomatics, Faculty of Medicine, RWTH Aachen, Pauwelsstr. 30, 52074 Aachen, Germany; 2 JARA-Translational Brain Medicine, Aachen, Germany; 3 Institute of Neuroscience and Medicine: JARA-Institute Brain Structure Function Relationship (INM 10), Research Center Jülich, Jülich, Germany

**Keywords:** Psychology, Human behaviour

## Abstract

Impulsive behavior tends to have a negative connotation in the sense that it is usually associated with detrimental or dysfunctional outcomes. However, under certain circumstances, impulsive behaviors may also have beneficial or functional outcomes. Dickman’s Impulsivity Inventory (DII) measures these two distinct aspects of impulsivity, namely, dysfunctional impulsivity (the tendency to act with less forethought than do most people which leads to difficulties) and functional impulsivity (the tendency to act with little forethought when the situation is optimal). In the present study, we translated the DII into German and validated the German version in a sample of 543 adults. The original 23-item model was considered unsuitable for the German version as suggested by fit indices of a confirmatory factor analysis. Exploratory factor analyses rather supported a 16-item version. Further psychometric analyses and inferential statistical analyses on the final German DII indicated its appropriateness for use in German-speaking populations and support a two-factor solution of the DII. Finally, exploratory analyses on the German DII suggest differential relationships between dysfunctional and functional impulsivity and self-reported lifestyle-related variables (smoking, alcohol usage, and sports behavior).

## Introduction

‘Impulsivity’ commonly refers to a range of behaviors which are often considered premature without foresight and irrespective of potential negative consequences. Since the term ‘impulsivity’ is used to describe numerous kinds of behavior, a variety of different definitions exist in the literature. The International Society For Research on Impulsivity defines impulsivity as “behavior without adequate thought, the tendency to act with less forethought than do most individuals of equal ability and knowledge, or a predisposition toward rapid, unplanned reactions to internal or external stimuli without regard to the negative consequences of these reactions”^1^. Common to almost all definitions is the reference to maladaptive behaviors with the potential for negative consequences^2^. Maladaptive expressions of impulsivity are frequently seen in psychiatric patient groups such as borderline personality disorder or attention-deficit/hyperactivity disorder^3–5^. But also in healthy populations, impulsive behavior and its consequences have a negative connotation. Self-report instruments designed to assess impulsivity are thus biased to measure negative outcomes^6^. And indeed, a multitude of studies has shown an association between high scores on such self-report measures and maladaptive behaviors in real life. For instance, elevated levels of self-reported impulsivity are found in reckless driving^7^, problematic internet use, nicotine smoking, and excessive alcohol use^8^.

However, evidence has also shown that impulsive tendencies in the sense that they reflect predispositions to act with little forethought may be beneficial. Early research on impulsive behavior has already shown that high impulsiveness can be beneficial for successful performance on information processing tasks when the time available for making a decision is extremely short^9^. This finding contradicted previous findings that high impulsive individuals are faster in their responses but also less accurate^9^. An unresolved question was thus whether the factor to respond rapidly and erroneously is the same factor that causes individuals to respond rapidly when this behavior is beneficial. Continuous work by Dickman (1990) has yielded a questionnaire that captures two distinct facets of impulsive behavior: functional and dysfunctional impulsivity^10^. The final version of Dickman’s Impulsivity Inventory (DII) comprises 23 items in total, 11 measuring aspects of functional impulsivity and 12 measuring aspects of dysfunctional impulsivity. According to Dickman (1990), dysfunctional impulsivity refers to the tendency to act without foresight in situations in which this behavior is not beneficial. In contrast, functional impulsivity refers to the tendency to make quick decisions with advantageous outcomes. These two independent factors have been differentially associated with various types of behaviors, among them lifestyle-related behaviors. For example, dysfunctional impulsivity on the DII has been shown to be related to smoking behavior^11^ and pathological gambling^12^. In contrast, functional impulsivity on the DII has been shown to be associated to greater perceptual processing speed^10^. Moreover, functional but not dysfunctional impulsivity seems to be related to greater risk-taking behavior when this behavior is optimal, leading to beneficial outcomes^13^. Greater functional impulsiveness has also been related to less cigarette craving suggesting that the DII may be useful in clinical contexts (e.g., for the identification of functional strengths of an individual). In addition, certain kinds of sports whose successful execution require quick decisions and a high level of concentration may also benefit from functional impulsiveness^14^. This seems to be particularly true for team sports as opposed to endurance sports^15^. Motor impulsivity, for instance, seems to have advantageous effects on the tactical performance of soccer players^16^. Conversely, Lage et al. reported a sub-facet of impulsivity (non-planning impulsivity) to be associated with more technical errors in handball games^17^, while attentional impulsivity was related to more fouls in this study. These contradicting results again suggest that different aspects of impulsivity exist and may be linked to different functional outcomes.

The DII has already been translated into several languages. According to our literature search, the DII is currently available in Dutch^18^, French^19^, Spanish^20^, Chinese^21^, and Brazilian Portuguese^6^. Across studies, the translated versions of the DII show good psychometric properties but some studies removed several items in order to obtain acceptable factor structures (e.g., ^6,18,20^). As already pointed out elsewhere^22^, Dickman conducted an exploratory factors analysis and retained the first two identified factors which mirrored his two-dimensional typology of impulsivity. Though some of the items cross-loaded onto both factors. This pertains especially to items 4, 7, and 22 and is reflected in some of the translation studies: item 4 (originally proposed to belong to the dysfunctional factor), for instance, cross-loaded onto both factors in the Spanish and the French version, whereas it loaded on the functional factor in the Brazilian and the Dutch version. It should be noted that this item had the lowest factor loading in Dickman’s original version and may, thus, not represent a good index of functional impulsivity. Likewise, item 7 and item 22 loaded on a different dimension than originally proposed in the Brazilian and the Chinese translation study, respectively. One of the discussed limitations of Dickman’s original study is that ratio of cases to items/estimated parameters is rather low and lower than the minimum recommended ratio of 10:1^23^. Moreover, that study, as well as many of the translation studies are based on university students and results may not be entirely generalizable to other populations.

To our knowledge, a German validation has not yet been published, although two pieces of scientific work state having used unpublished German versions of the DII^24,25^. The use of unpublished, potentially differing questionnaires or inaccessible questionnaires may be problematic. It is not known whether the psychometric properties of these unpublished questionnaires were determined and whether they are acceptable. As outlined before, there seem to be problems with some of the DII items and these problems should be analyzed in a translation and validation study. Interpretation of data based on questionnaires, which may not be reliable or where single items may not represent the construct they are supposed to measure may lead to biased interpretations of associated results. The fact that some unpublished German DII versions circulate and are used in research, however, stresses the need for a validated German DII and emphasizes that its use is warranted. Some authors who investigate impulsivity in German-speaking populations even call for questionnaires that assess functional aspects of impulsivity^26,27^.

In conclusion, studies that have used the DII before could show practical significance between behavioral measures and DII scores. Especially the functional subscale may be employed for the identification of individual strengths which may be useful in clinical applications. To our knowledge, a validated German version is so far not available but it has been noted that impulsivity work might benefit from instruments that assess functional impulsivity.

Therefore, the present study aimed to translate the DII into German and to validate the translation using an appropriate-sized, diverse sample. In addition to translating and validating a German DII, we aimed to investigate the relationship between functional and dysfunctional impulsivity and lifestyle-related behaviors. Specifically, we hypothesized that dysfunctional but not functional DII scores were related to smoking and drinking behavior and that functional DII scores were higher in individuals performing team sports compared to individuals performing endurance sports.

## Methods

All procedures were in accordance with the Declaration of Helsinki and were approved by the University’s Ethics Committee (Ethics Committee at the RWTH Aachen Faculty of Medicine, internal reference number: EK 312/20). The study including hypotheses was preregistered prior to data collection (osf.io/45crz).

### Subjects

Initially, 745 individuals have started the survey but 202 participants had to be excluded as they did not answer all DII items. In total, 543 adults completed the online survey. Their age ranged between 18 and 82 years (*M* = 35.59, *SD* = 14.62). Further demographic information of participants may be found in Table 1.

**Table 1:** Participant characteristics.

	Group	*n* (%)
Gender	Females	357 (65.75)
	Males	185 (34.07)
	Diverse	1 (0.18)
Education level	Higher education degree	294 (54.14)
	Higher education entrance qualification	126 (23.20)
	Vocational qualification	83 (15.29)
	Secondary school graduation	40 (7.37)

### Procedure

A translation and adaptation process was performed according to international guidelines^28^. First, the English DII^10^ was translated into German by two native German speakers with a high proficiency in English. Differences between the two translations were debated in a discussion moderated by a third person. The resulting version was back-translated into English by a native bilingual of English and German language. The German version and the back-translation were proofread and adapted—in consultation with the two initial translators—by another native English speaker with a high proficiency in German. Finally, the final German DII survey was made publicly available via the web application software SoSci-Survey (https://www.soscisurvey.de).

Survey duration was approximately 10 min. The survey was spread through various mailing lists. We contacted the psychology departments of the universities of Cologne, Osnabrück, Hagen, and Aachen, asking them to forward our survey to their students. To not only rely on students, the survey was sent via the employee distribution list of ASB (Arbeiter–Samariter–Bund) and Pirmasens Hospital. Finally, friends and relatives of the authors were contacted, some of whom forwarded the survey to their colleagues via the distribution lists of their employers. Participants had to provide informed consent in order assess the survey. No specific inclusion or exclusion criteria were required for participation. Participants were not individually reimbursed for study participation but had the opportunity to win one out of 20 Amazon vouchers (of 10 € each). In order to assess the test-rest reliability of the German DII, we asked participants to indicate whether or not they were willing to participate in the survey a second time. Those who were willing to participate twice received an invitation to the second survey four weeks (28 days) after completing the first survey. The period of data collection of the initial survey was from mid-September 2020 to mid-November 2020. The period of data collection of the follow-up survey was from mid-October 2020 to mid-December 2020.

### Instruments

#### Dickman impulsivity inventory (DII)

The original DII consists of 23 self-report items and was designed two measure functional impulsivity (11 items) and dysfunctional impulsivity (12 items). It is based on a dichotomous answer format (true/false). However, instruments employing Likert-like answer formats have been shown to yield better psychometric properties compared to dichotomous scales^29,30^. We, therefore, adopted a five-point Likert-like response format (totally disagree, disagree, neutral, agree and totally agree), similar to the Brazilian DII^6^. For comparability, we maintained the same item order used in previous translation studies (see Supplementary Information 1, Supplementary Table S1), proposed by Claes et al.^18^.

#### Barratt Impulsiveness Scale-11

The Barratt Impulsiveness Scale (BIS-11; Patton et al.^31^) is a self-report instrument designed to assess trait impulsiveness. It comprises 30 items, which are to be rated on a 4-point Likert-like scale reflecting the frequency of occurrence. The German BIS-11^32^ was administered in the current study in order to assess convergent and discriminant validity of the German DII. The internal consistency (Cronbach’s alpha) of the German BIS-11 has been reported to range from 0.69 for the general population to 0.83 for psychiatric patients^32^. Cronbach’s alpha of the BIS-11 total score for the current sample was 0.78.

#### Demographics and lifestyle-related questions

Participants were asked to report their age (years), gender (male, female, diverse), and education. Lifestyle-related questions comprised questions on smoking (smoker, non-smoker, ex-smoker, occasional smoker^33^, and drinking habits, including questions from the Fagerström Test for Nicotine Dependence (FTND; Heatherton et al.^34^) and the Alcohol Use Disorders Identification Test (AUDIT; Saunders et al.^35^). Questions on sports behavior included whether participants performed any kind of sports at all (yes/no), how often they performed sports, and which kind of sports they performed.

### Data analyses

Confirmatory factor analysis (CFA) and exploratory factor analysis (EFA) were carried out to assess the construct validity of the German DII. First, the Kaiser–Meyer–Olkin (KMO) Measure of Sampling Adequacy and Bartlett’s Test of Sphericity were used to assess the suitability of the data for factor analysis. The KMO Measure of Sampling Adequacy indicates the proportion of variance in all variables that might be caused by underlying factors. Values of KMO ≥ 0.8 indicate appropriateness of the sampling for factor analysis. A significant Bartlett's test of sphericity rejects the hypothesis that items in question are unrelated and therefore unsuitable for structure detection.

Subsequent to the assessment of suitability of the data for data reduction techniques, a CFA was conducted in order to investigate whether the factor structure originally proposed by Dickman (two-factor solution) was a good fit to our data. We chose the unweighted least squares (ULSMV) estimation method to account for the ordinal nature of the response scale^36,37^. The model was fit to the variance–covariance matrix of our data. We first fit the model assuming correlated factors. Since correlations were negligible, a second CFA was carried out assuming uncorrelated factors. As the results of our CFA models were unsatisfactory, EFA were carried out.

To determine the type of rotation, we first ran an EFA using an oblique (Oblimin) rotation to calculate inter-factor correlation. Due to a low correlation, the subsequent EFAs were all done using orthogonal (Varimax) rotations. All EFAs were done with the correlation matrix of the data using the unweighted least squares factor extraction method.

Measures of fit assessing the models’ goodness of fit^38,39^ included the Tucker Lewis Index (TLI; values > 0.90 indicating good fit), and the root mean square error of approximation (RMSEA; values of < 0.06 indicating close fit) and the Standardized Root Mean Residual (SRMR; values of < 0.08 indicating good fit). For the CFA, we also considered the confirmatory factor index (CFI; values > 0.95 indicating good fit). Factor loadings of at least │0.3│ were considered to meet the minimal level for further interpretation^23^.

Internal consistency of the final model was assessed by Cronbach’s alpha with values of *α* ≥ 0.7 considered as acceptable. Test–retest reliability was assessed for a subsample of 101 participants who completed the German DII twice. On average, the questionnaires were completed 29.34 days apart (Median = 28.0, *SD* = 3.80, min = 28.0, max = 52.0). Values of *r* ≥ 0.7 were considered acceptable. In order to assess convergent and discriminant validity, the two DII subscales were correlated with the BIS-11.

Finally, we carried out exploratory analyses on the German DII and self-reported lifestyle-related variables. Specifically, we carried out Kruskal–Wallis tests (due to residual non-normality) with smoking status (current smoker, ex-smoker, occasional smoker, non-smoker) as independent variable and dysfunctional and functional DII scores as dependent variables. Unpaired two-samples Wilcoxon tests on the DII scales were conducted between individuals who indicated to perform teams sports and individuals who indicated to perform other kinds of sports. Finally, we performed Pearson correlations between total AUDIT scores and DII scores. For these inferential statistical analyses, the significance level was set at an alpha level of 5%. All analyses were conducted in RStudio Team (2021)^40^. We used the lavaan package for CFA^41^ and the psych package^42^ for EFA.

## Results

### Factor analyses

A KMO value of 0.86 and a significant Bartlett's Test of sphericity (*X*^2^ (253) = 872.02, *p* < 0.001) indicated that the data was suitable for factor analysis. The structure, as proposed by Dickman (1990) was tested for the German DII through CFA. The two-factor solution assuming correlated and uncorrelated factors, however, indicated poor fit across all indices (see Table 2). Therefore, EFA were carried out. In a consecutive procedure, in the course of fitting 3 EFA models, we removed 7 items due to inadequate fit of two first EFA models. A third and final model was deemed acceptable and indicative of good fit (see Table 2). The exact procedure of fitting the EFA models, corresponding fit indices, and reasons for item exclusion may be found in Supplementary Information 2. The main reasons for item exclusion were that some of the items did not load onto any factor (i.e., had loadings below │0.3│) whereas other items loaded on a different factor than originally proposed.

**Table 2:** Fit indices for factor analysis models.

	Number of items	CFI (> 0.95)	TLI (> 0.90)	SRMR (< 0.08)	RMSEA (< 0.06)
CFA (correlated factors assumed)	23	0.76	0.73	0.115	0.112
CFA (uncorrelated factors assumed)	23	0.76	0.73	0.117	0.113
Final EFA	16	–	0.90	0.04	0.06

*CFA* confirmatory factor analysis, *EFA* exploratory factor analysis, *CFI* Comparative Fit Index, *TLI* Tucker-Lewis Index, *SRMR* Standardized Root Mean Residual, *RMSEA* Root Mean Square Error of Approximation.

**Figure 1:**
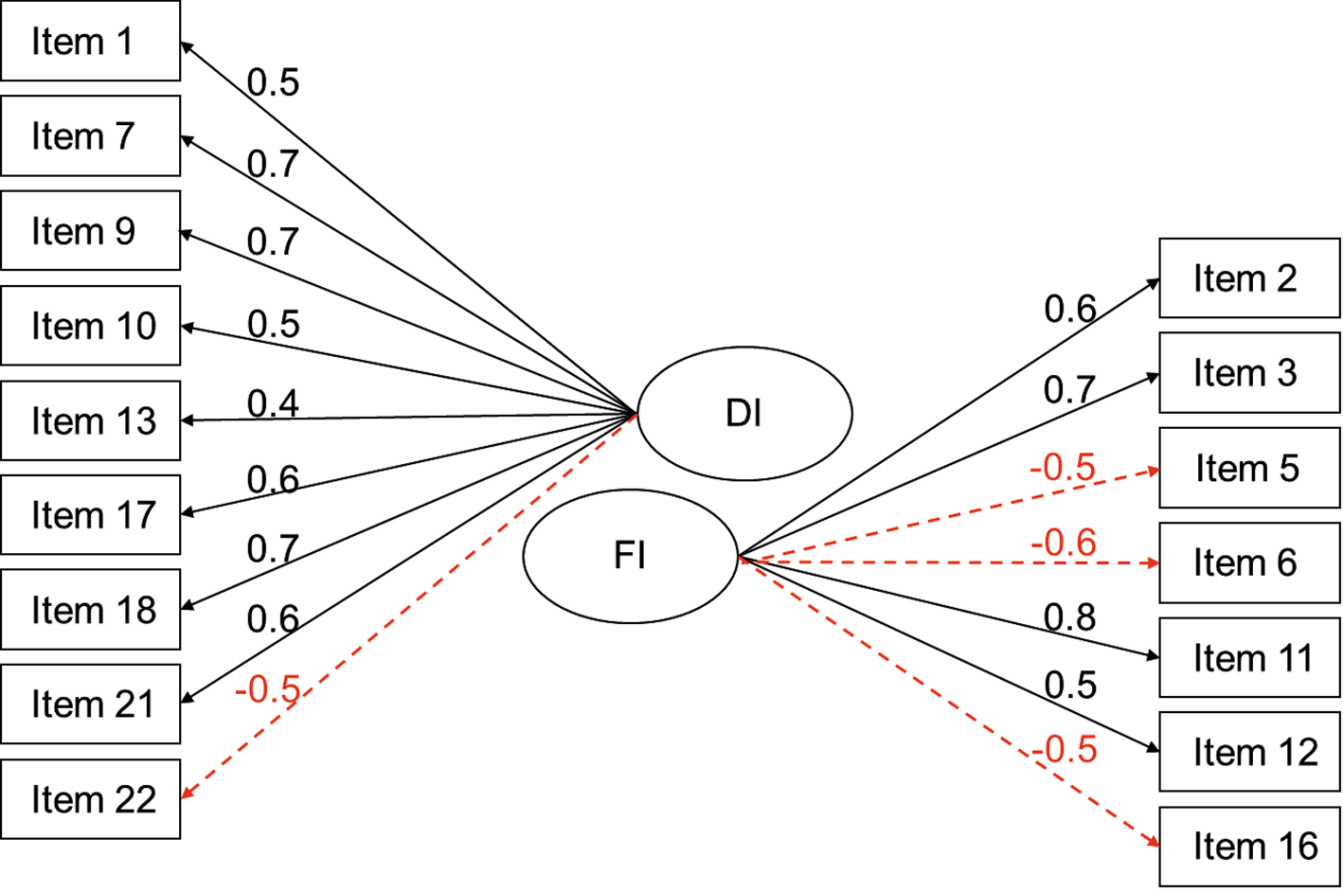
Factor loadings for the dysfunctional (DI) and functional (FI) impulsivity subscale derived from the final exploratory factor analysis model. Item order as proposed by Claes et al.^18^. Negative values (in red) suggest a negative linear association between the latent variable and the observed item.

Figure 1 presents our final model. Negative factor loadings suggest a negative linear association between the latent variable and the observed item. Negative loadings are typically related to differences in phrasing (i.e., negatively versus positively phrased questionnaire items) within one scale. Either items with the positive or the negative loadings must have their data values reversed. We reversed items with negative loadings for the dysfunctional DII scale, such that higher total scores on the dysfunctional DII reflect higher levels of impulsiveness. In order for summary scores of the functional scale to also reflect greater impulsiveness, we had to reverse items with positive factor loadings on this scale.

### Reliability analyses

Internal consistency of the final 16-item version of the German DII was acceptable (for functional impulsivity, *α* = 0.78) to good (for dysfunctional impulsivity, α = 0.80). Test–retest reliabilities were also satisfactory with *r* = 0.77 for the dysfunctional and *r* = 0.84 for the functional DII scale. In addition, paired samples t-tests indicated that total scores on the dysfunctional (*t*(100) = − 1.36; *p* = 0.18) and on the functional (*t*(100) = − 0.71, *p* = 0.48) DII scales did not differ between the two time points.

### Discriminant and convergent validity

Pearson correlations were carried out between the DII subscales and the BIS-11. The BIS-11 showed a significant positive correlation with the dysfunctional DII subscale (*r* = 0.67, *p* < 0.001), but no relation to the functional DII subscale (*r* = − 0.05, *p* = 0.29). Furthermore, the two DII scales showed no relation to each other (*r* = − 0.04, *p* = 0.41). A comparison between psychometric properties of the final German DII and psychometric properties identified by previous translations of the DII may be found in Table 3.

**Table 3:** Comparison of DII psychometric properties across validation studies.

DII version	Internal consistency (Cronach’s alpha)		Test–retest reliability		Correlation between FI and DI scale (*r*)
	DI	FI	DI	FI	
German	0.80	0.78	0.77 (r)	0.84 (r)	− 0.04
American	0.85	0.74	NA	NA	0.23
Brazilian	0.75	0.73	0.85 (ICC)	0.89 (ICC)	− 0.031
Chinese	0.75	0.68	NA	NA	0.250
Dutch	0.84	0.76	NA	NA	− 0.02
French	0.80	0.76	0.82 (males) 0.53 (females)	0.44 (males) 0.70 (females)	NA
Spanish	0.76	0.78	0.765 (r)	0.757 (r)	0.28

*DI* dysfunctional impulsivity subscale, *FI* functional impulsivity subscale, *NA* not available, *r* Pearson correlation, *ICC* intraclass correlation coefficient.

### Relationship with lifestyle-related variables

Results of Kruskal–Wallis tests indicated that smoker status had a significant effect on dysfunctional (*χ*^2^ (3) = 10.1, *p* = 0.018, η^2^ = 0.013), but not on functional DII scores (*χ*^2^ (3) = 1.61, *p* = 0.66). The associated Bonferroni-adjusted post-hoc test (Dunn’s test) indicated that current smokers had significantly higher dysfunctional DII scores compared to non-smokers (see Table 4). No further post-hoc tests were significant. Among smokers, higher scores on the Fagerström Test for Nicotine Dependence (FTND) were weakly related to dysfunctional impulsivity (*r* = 0.24, *p* = 0.04) but not to functional impulsivity (*r* = − 0.03, *p* = 0.80).

**Table 4:** DII scores as a function of smoking status and sports behavior.

	*n* (%)	DI *M* (*sd*)	FI *M* (*sd*)
**Smoking status**			
Non-smokers	345 (63.54)	19.33 (5.23)^a^	21.96 (4.97)
Ex-smokers	78 (14.36)	20.44 (5.75)	22.37 (4.59)
Smokers	71 (13.08)	21.25 (5.39)^a^	22.25 (4.90)
Occasional smokers	49 (9.02)	19.71 (5.11)	22.76 (4.76)
**Sports behavior**			
Teams sports	63 (11.60)	19.52 (4.84)	23.73 (4.36)^b^
Other kind of sports	302 (55.61)	19.37 (5.38)	22.13 (5.01)^b^

*DI* dysfunctional impulsivity, *FI* functional impulsivity.

^a^Significantly different from each other, *p* = 0.018 (Dunn’s post-hoc test; Bonferroni-adjusted).

^b^Significantly different from each other, *p* = 0.015 (Wilcoxon-Test).

Among participants who indicated to perform sports regularly, those who indicated to perform team sports had higher functional DII scores compared to individuals who performed other kinds of sports (*W* = 7667, *p* = 0.015). Groups did not differ on the dysfunctional DII subscale (*W* = 9075.5, *p* = 0.57).

Finally, AUDIT total scores were not related to functional impulsivity (*r* = 0.07, *p* = 0.10) but were related to dysfunctional impulsivity: Namely, higher scores on the AUDIT showed a small positive correlation with dysfunctional DII scores (*r* = 0.21, *p* < 0.001).

In order to correct for multiple comparisons, we re-evaluated our results using an adjusted *p*-value of 0.017 [we divided the original *p*-value of 0.05 by 3 as per independent hypothesis: effects of (1) smoking, (2) sports behavior, and (3) alcohol consumption on DII scores]. The effect of smoking status remained marginally significant. The correlation between FTND scores and dysfunctional DII scores failed to reach significance when considering the adjusted p-value. The findings on the significant association between sports behavior and functional impulsivity and AUDIT scores and dysfunctional impulsivity remained significant.

## Discussion

Impulsive behavior is not only restricted to dysfunctional outcomes and behavioral components but reveals functional aspects as well. Dickman’s Impulsivity Inventory (DII) reflects this fact in the assessment of both, functional and dysfunctional impulsivity^10^. The current study aimed to translate the original English DII into the German language and to validate the translation. In addition, we investigated the relationship between both functional and dysfunctional impulsivity and lifestyle-related behaviors.

Results of our study support a 16-item version rather than the original 23-item version of the DII for the German population. Our final factor structure with nine items on the dysfunctional scale and seven items on the functional scale demonstrated good psychometric properties. The fact that we had to remove items in order to achieve satisfactory psychometric indices is in line with a revalidation of the English DII. This study investigated psychometric properties of the dysfunctional subscale resulting in a 9-item dysfunctional model^22^. Moreover, previous DII translation studies also removed items in order to achieve satisfactory psychometric properties. Similar to our study, the Brazilian and Dutch translation study found large factor loadings for item 4 for the functional scale, whereas it was considered to belong to the dysfunctional scale in the original DII. The Spanish and the French translation found almost equal loadings for both subscales for item 4. As already recognized previously^18^, this item had the lowest loading in Dickman’s original study and may not be considered as a good indicator of functional impulsivity. Furthermore, in our study, items 8 and 23 showed no distinct loadings on either of the two scales, similar as reported by Claes et al.^18^. Furthermore, our results showed that three items (i.e., 15, 19, 20), we found loaded on a third factor (i.e., neither on the functional nor on the dysfunctional factor). Examination of items loading on this factor revealed that all of these items describe the ability to express or verbalize one's thoughts quickly or to think quickly (e.g., “Most of the time, I can put my thoughts into words very rapidly”). Thus, they differ from other original FI items, which rather measure decisional impulsivity (e.g., “I am good at taking advantage of unexpected opportunities, where you have to do something immediately or lose your chance”). Regarding item 15, inconsistencies were also reported by the Brazilian study in which this item showed no distinct loadings on any factor. The Brazilian study only considered a two-factor solution. Therefore, it is unknown how their results would have looked like if they also considered a three-factor solution. It may be the case that a three-factor solution would have revealed a different pattern.

Additional psychometric analyses underscore the appropriateness for use of our final version: Cronbach’s alpha, an index of internal consistency, of *α* = 0.78 and *α* = 0.80 for the functional and dysfunctional DII respectively, are acceptable to good and are comparable to alpha values reported by previous DII translation studies (see Table 3). High test–retest reliability scores demonstrate that the German DII is reliable over time, indicating that it measures trait characteristics. Importantly, the subscales of the DII are thought to be independent factors and to measure distinct behaviors. In our study as well as in the Dutch and Brazilian translations, the two scales were found to be unrelated. In contrast, other translation studies and the original American DII reported low positive associations between the dysfunctional and the functional DII scales (see Table 3), probably due to the inclusion of items not closely associated with functional impulsivity (quick verbalization). In addition, we found no association between the functional subscale of the DII and total BIS-11 scores, while we found a positive relationship between the dysfunctional subscale and the BIS-11. The BIS-11 is the most widely used self-report measure of impulsivity^43^ and is thought to tap into dysfunctional aspects of it.

Overall, the German version of the DII showed satisfactory psychometric properties and may be used in further studies.

In addition to its psychometric soundness, the DII seems to be differentially associated with different lifestyle-related behaviors. Specifically, smoking behavior and hazardous alcohol consumption were related to dysfunctional but not to functional impulsivity. Conversely, participants of our study who indicated to perform team sports reported higher functional, but not dysfunctional impulsivity compared to individuals who indicated to perform endurance sports. These results are in line with previous studies demonstrating an association between smoking^44^ as well as hazardous alcohol consumption^45^ and self-reported impulsivity, including self-reported dysfunctional impulsivity on the DII^11,46^. Moreover, previous studies have already highlighted the beneficial aspects of impulsive behavior in team sports such as soccer^16^ which require the ability to make rapid decisions while maintaining a high level of concentration, one of the positive aspects of (functional) impulsiveness.

In sum, psychometric results of the DII, as well as results on associative relationships between the DII and self-reported behaviors, clearly support the notion that the functional and the dysfunctional DII subscales are independent factors and are differentially related to diverse behavioral outcomes. Beyond that, future research may assess whether functional manifestations of impulsive behavior have the potential to serve as a resource to refrain from behaviors typically regarded as dysfunctional. For example, Pitts and colleagues reported a negative relationship between functional impulsivity on the DII and cigarette craving in smokers^11^. Insights gained from such studies may be of interest to clinical or translational research focusing on resources or strategies to improve health.

Our work further supports the work by Dickman^10^ that impulsivity is a non-unitary construct^47,48^. The numerous experimental paradigms and self-reports used in psychological research for the assessment of impulsivity are unlikely to all measure aspects of the same underlying psychometric factor. In this line, several studies failed to show a relationship between self-reported impulsivity and experimental measures of impulsivity^49^. Recent voices even argue that impulsivity may not be considered as a psychological construct at all as it does not fulfill basic requirements of a psychological construct^50^. The DII may contribute to filling the gap between self-reported impulsivity and experimental work on it. Some experimental work clearly identified beneficial outcomes associated with behavior traditionally operationalized as impulsive^13^. In addition, researchers state that questionnaires—so far lacking in the German literature—which assess functional impulsivity may augment their experimental research^26,27^.

Nevertheless, the results from this study should be interpreted considering several limitations. First, our sample comprised rather highly educated participants, with more than half of the sample (54.14%) holding a higher education degree and 23.20% indicating they had a higher education entrance qualification. Only 7.37% of participants indicated they had a secondary school graduation (other than higher education entrance qualification) or lower, and 15.29% indicated they hold a vocational qualification. Most psychological questionnaires have been developed in populations with rather high levels of education (often using students as convenience samples). However, they may produce false-positive results when applied in populations with rather low levels of education^6^. Even though we tried to reach participants with diverse educational backgrounds, we mainly reached participants with high levels of education and it was not possible to perform any sub-group factor analyses. Therefore, the German DII may be tested in other populations to increase generalizability. Likewise, the German DII may be tested in different populations with psychiatric diseases. Dysfunctional impulsivity is present in many psychiatric diseases and impulsivity measures have been shown to produce differing psychometric properties for different psychiatric groups^32^. As outlined above, it may also be of interest to assess functional aspects of impulsivity in clinical contexts to identify individual strengths; for this normative data in different psychiatric samples is necessary.

A further limitation of the current study pertains to the assessment of convergent validity. We assessed discriminant and convergent validity using summary scores of the BIS-11. This instrument is a commonly used measure of (dysfunctional) impulsivity and shows adequate psychometric properties in clinical populations. For the general population, the German BIS-11, however, shows a questionable internal consistency (Cronbach’s alpha = 0.69)^32^. Furthermore, research on the BIS-11 has shown that its summary score may not reflect a common latent trait of impulsivity^51^. These findings on the BIS-11 may reflect the heterogeneity of impulsivity. Therefore, our results regarding convergent and discriminant validity may be interpreted with caution. Nevertheless, we could show a significant association between dysfunctional, but not functional DII scores and BIS-11 summary scores, highlighting those dysfunctional aspects of impulsivity captured by the DII may be associated with aspects of impulsivity assessed by the BIS-11. Future studies may further investigate the convergent validity of the German DII, assessed by other self-report impulsivity questionnaires such as the UPPS-P Impulsive Behaviour Scale^52^ which also assesses different aspects of impulsivity but does not recommend a summary score. Moreover, the functional DII scale may be further investigated with respect to the relationship between functional DII scores and behavioral responses on laboratory tasks assessing impulsivity. This brings us to another limitation of the present study: all our measures (the DII itself as well as variables/constructs we related it to) were self-report measures. This may have introduced common-method variance biasing the association between the DII and other variables used to assess the DII’s reliability/validity. To address this problem, laboratory task measures of impulsivity would be necessary. Regarding dysfunctional aspects of impulsivity, there appears to be little overlap between self-reported and behavioral impulsivity (assessed through laboratory tasks)^50^. However, future studies may benefit from collecting objective evidence to further validate the functional DII scale.

Furthermore, we changed the item answering option from dichotomous to a five-point Likert scale. Comparing our final model to previous models of the DII which used a dichotomous scale may not be optimal. Nonetheless, research has shown that response formats with only two options have lower reliability than Likert-like response scales^29,53,54^. Commonly, four to seven answering alternatives seem to produce the most reliable results^29,54^. Moreover, the percentage of variance explained by latent factors seems to decrease as the number of answering alternatives decreases^29^. Indeed, our model performed well or similarly in comparison to previous DII models (e.g., regarding internal consistency).

Finally, our data was collected via an online-survey and it is not clear if this method compares with paper–pencil administration. Related to the nature of online-surveys, we had to exclude 202 participants due to incomplete DII data. This reduction in the number of cases possibly limits representativeness and some response-bias may be at play. However, to the best of our knowledge, the present study provides acceptable to good psychometric properties of a German DII version based on a large, randomly selected sample.

In conclusion, the 16-item German DII seems to be a valid measure of functional and dysfunctional impulsivity, which appear to be two independent factors. The translation and validation process led to the exclusion of seven DII items to achieve satisfactory psychometric results. The factor structure, however, did not change in the course of this process. Our results suggest that the German DII is a valid and reliable instrument for use in German-speaking samples but may be further evaluated in different populations such as in psychiatric groups.

## Supplementary Information

Supplementary Information 1.

Supplementary Information 2.

## Data Availability

The datasets generated during and analyzed during the current study are available from the corresponding author on reasonable request.
