# Supplementary material for: Adaptation and validation of a German version of the Dickman impulsivity inventory for the assessment of functional and dysfunctional impulsivity
Source: Sci Rep. 2021 Dec 2;11:23310. doi: 10.1038/s41598-021-02775-1 (PMC8639757; doi:10.1038/s41598-021-02775-1)
Supplement: Supplementary file 1 — Supplementary Information 1. [file 41598_2021_2775_MOESM1_ESM.pdf]

Supplementary Material 1: original and translated DII items

Adaptation and Validation of a German Version of the Dickman Impulsivity Inventory for the Assessment of Functional and Dysfunctional Impulsivity

Philippa Hüpen[1,2*], Alina T. Henn[1], Ute Habel[1,3]

[1] Department of Psychiatry, Psychotherapy and Psychosomatics, Faculty of Medicine, RWTH Aachen, Aachen, Germany
[2] JARA - Translational Brain Medicine, Aachen, Germany
[3] Institute of Neuroscience and Medicine: JARA-Institute Brain Structure Function Relationship (INM 10), Research Center Jülich, Jülich, Germany

*Correspondence to:
Philippa Hüpen
Department of Psychiatry, Psychotherapy und Psychosomatics, University Hospital RWTH Aachen
Pauwelsstr. 30
52074 Aachen
Germany
Tel.: +49 241 80 89730
Email: rhuepen@ukaachen.de

Table S1

Original items of Dickman's Impulsivity Inventory and the corresponding German translation

Item order proposed by Claes et al. (2000)	Subscale	Original version (Dickman, 1990)	German version
1	D	Often, I don't spend enough time thinking over a situation before I act.	Ich nehme mir oft nicht genug Zeit, über eine Situation nachzudenken, bevor ich handle.
2	F	I try to avoid activities where you have to act without much time to think first	Ich versuche Aktivitäten zu vermeiden, bei denen man ohne viel Zeit zum Nachdenken handeln muss.
3	F	I don't like to make decisions quickly, even simple decisions, such as choosing what to wear, or what to have for dinner.	Ich mag es nicht gerne schnell Entscheidungen zu treffen, auch keine einfachen Entscheidungen, wie z.B. was ich anziehen oder zu Abend essen soll.
4	D	I enjoy working out problems slowly and carefully.	Ich löse Probleme gerne langsam und sorgfältig.
5	F	I am good at taking advantage of unexpected opportunities, where you have to do something immediately or lose your chance.	Ich bin gut darin unerwartete Gelegenheiten für mich zu nutzen, bei denen man sofort handeln muss oder seine Chance verliert.
6	F	I would enjoy working at a job that required me to make a lot of split-second decisions.	Mir würde es Freude bereiten, einer Arbeit nachzugehen, bei der ich viele Entscheidungen in Sekundenbruchteilen treffen muss
7	D	I often make up my mind without taking the time to consider the situation from all angles.	Ich treffe oft Entscheidungen, ohne mir die Zeit zu nehmen, die Situation aus allen Blickwinkeln zu betrachten.
8		I have often missed out on opportunities because I couldn't make up my mind fast enough.	Ich habe schon oft Chancen verpasst, weil ich mich nicht schnell genug entscheiden konnte.
9	D	I often say and do things without considering the consequences	Ich sage und tue oft Dinge, ohne die Konsequenzen zu berücksichtigen.
10	D	I frequently make appointments without thinking about whether I will be able to keep them.	Ich vereinbare häufig Termine, ohne darüber nachzudenken, ob ich diese einhalten kann.
11	F	I am uncomfortable when I have to make up my mind rapidly.	Ich fühle mich unwohl, wenn ich mich schnell entscheiden muss.
12	F	I don't like to do things quickly, even when I am doing something that is not very difficult.	Ich mag es nicht Dinge schnell zu erledigen, selbst wenn ich etwas tue, das nicht sehr schwierig ist.
13	D	I frequently buy things without thinking about whether or not I can really afford them.	Ich kaufe häufig Dinge, ohne darüber nachzudenken, ob ich sie mir wirklich leisten kann.
14	D	I am good at careful reasoning	Ich bin gut darin, logisch zu Argumentieren.
15	F	I like to take part in really fast-paced conversations, where you don't have much time to think before you speak.	Ich beteilige mich gerne an regen Unterhaltungen, bei denen man nicht viel Zeit zum Nachdenken hat, bevor man spricht.
16	F	I like sports and games in which you have to choose your next move very quickly.	Ich mag Sportarten und Spiele, bei denen man seinen nächsten Zug schnell wählen muss.

17	D	Many times the plans I make don't work out because I haven't gone over them carefully enough in advance.	Meine Pläne gehen oftmals nicht auf, weil ich sie im Vorfeld nicht sorgfältig genug durchdacht habe.
18	D	I often get into trouble because I don't think before I act.	Ich gerate häufig Schwierigkeiten, weil ich nicht nachdenke, bevor ich handle.
19	F	Most of the time, I can put my thoughts into words very rapidly.	Most of the time, I can put my thoughts into words very rapidly.
20	F	People have admired me because I can think quickly.	Leute bewundern mich, weil ich schnell denken kann.
21	D	I will often say whatever comes into my head without thinking first.	Ich sage oft, was mir in den Sinn kommt, ohne darüber nachzudenken.
22	D	Before making any important decision, I carefully weigh the pros and cons.	Bevor ich eine wichtige Entscheidung treffe, wäge ich sorgfältig Vor- und Nachteile ab.
23	D	I rarely get involved in projects without first considering the potential problems.	Ich beteilige mich selten an Projekten, ohne vorher mögliche Probleme zu berücksichtigen.

Note. F = functional impulsivity subscale; D = dysfunctional impulsivity subscale.
Participants responded on a 5-point Likert scale: 1 = totally agree (stimme völlig zu), 2 = agree (stimme zu), 3 = neutral (stimme weder zu noch nicht zu), 4 = disagree (stimme nicht zu), 5 = totally disagree (stimme überhaupt nicht zu).

References

Claes, L., Vertommen, H., & Braspenning, N. (2000). Psychometric properties of the Dickman Impulsivity Inventory. *Personality and Individual Differences*, *29*(1), 27–35. https://doi.org/10.1016/S0191-8869(99)00172-5

Dickman, S. J. (1990). Functional and Dysfunctional Impulsivity: Personality and Cognitive Correlates. *Journal of Personality and Social Psychology*, *58*(1), 95–102. https://doi.org/10.1037/0022-3514.58.1.95
