# Supplementary material for: Adaptation and validation of a German version of the Dickman impulsivity inventory for the assessment of functional and dysfunctional impulsivity
Source: Sci Rep. 2021 Dec 2;11:23310. doi: 10.1038/s41598-021-02775-1 (PMC8639757; doi:10.1038/s41598-021-02775-1)
Supplement: Supplementary file 2 — Supplementary Information 2. [file 41598_2021_2775_MOESM2_ESM.pdf]

Supplementary Material 2: exploratory factor analyses

Adaptation and Validation of a German Version of the Dickman Impulsivity Inventory for the Assessment of Functional and Dysfunctional Impulsivity

Philippa Hüpen[1,2*], Alina T. Henn[1], Ute Habel[1,3]

[1] Department of Psychiatry, Psychotherapy and Psychosomatics, Faculty of Medicine, RWTH Aachen, Aachen, Germany
[2] JARA - Translational Brain Medicine, Aachen, Germany
[3] Institute of Neuroscience and Medicine: JARA-Institute Brain Structure Function Relationship (INM 10), Research Center Jülich, Jülich, Germany

*Correspondence to:
Philippa Hüpen
Department of Psychiatry, Psychotherapy und Psychosomatics, University Hospital RWTH Aachen
Pauwelsstr. 30
52074 Aachen
Germany
Tel.: +49 241 80 89730
Email: rhuepen@ukaachen.de

Since we could not retain the original factor structure of the DII through CFA, we carried out EFA. The number of potential factors was determined by a parallel analysis and a scree plot. These methods indicated a two- or three-factor solution. Since our aim was to replicate a two-factor structure with a functional and a dysfunctional factor, our focus was on two-factor models. However, in cases we had to exclude items, we verified reasons for exclusion by additionally inspecting a potential three-factor solution.

In order to determine the type of rotation, we first ran an EFA using an oblique (Oblimin) rotation to calculate inter-factor correlation. Due to a low correlation coefficient (r = .05), we ran an additional model using orthogonal (Varimax) rotation. The EFA models, based on all original DII items (EFA1) were considered not suitable in terms of fit indices (see Table S2). All subsequent EFAs were done using orthogonal (Varimax) rotations. Four items were excluded in the next analysis (EFA2): item 4 loaded on the functional impulsivity factor (in a two-factor as well as a three-factor model), whereas it was considered to belong to the dysfunctional factor in the original DII. Item 23, which was considered to belong to the dysfunctional impulsivity factor in the original DII did not load on any factor in a two-factor model, whereas it loaded on the functional impulsivity factor in a three-factor model. Finally, items 8 and 14 loaded on the functional and the dysfunctional factor in both models.

Results of the second EFA (EFA2) were still unsatisfactory. Although all items showed factor loadings above .30 on only one factor and on the same factor as proposed by the original DII, goodness of fit values were still unsatisfactory for a two-factor solution (see Table S2). Similarly, a three-factor model on the same items was also considered unsatisfactory. Therefore, we further investigated items of the third factor. All items exclusively loading on this factor were items originally proposed to belong to the functional impulsivity factor. When examining items with high loadings on this factor (3 items), it became apparent that these items had a different quality compared to other items of the

functional impulsivity subscale (see Discussion). After removing these items, goodness of fit values for our final EFA were satisfactory.

Table S2

Fit indices for factor analysis models assuming two factors.

	Number of items	CFI (> .95)	TLI (> .90)	SRMR (< .08)	RMSEA (< .06)	90% CI for RMSEA
CFA (correlated factors assumed)	23	.76	.73	.115	.112	.11-.12
CFA (uncorrelated factors assumed)	23	.76	.73	.117	.113	.11-.12
EFA1 (two factors; Oblimin rotation)	23	-	.79	.06	.07	.07-.08
EFA1 (two factors; VARIMAX rotation)	23	-	.79	.06	.07	.07-.08
EFA1 (three factors VARIMAX rotation)	23	-	.90	.04	.05	.04-.06
EFA2 (two factors VARIMAX rotation)	19	-	.81	.06	.07	.07-.08
EFA2 (three factors VARIMAX rotation)	19	-	.91	.04	.05	.04-.06
Final EFA (VARIMAX rotation)	16	-	.90	.04	.06	.05-.07

Note. CFA = confirmatory factor analysis; EFA = exploratory factor analysis; CFI = Comparative Fit Index; TLI = Tucker-Lewis Index; SRMR = Standardized Root Mean Residual; RMSEA = Root Mean Square Error of Approximation; CI = confidence interval.
